# Reliability of kettlebell swing one and five repetition maximum

**DOI:** 10.7717/peerj.14370

**Published:** 2022-11-21

**Authors:** James A. Ross, Justin W. L. Keogh, Christian Lorenzen

**Affiliations:** 1 School of Behavioural and Health Sciences, Australian Catholic University, Melbourne, Victoria, Australia; 2 Faculty of Health Sciences and Medicine, Institute of Health & Sport, Bond University, Gold Coast, Queensland, Australia; 3 Manipal Academy of Higher Education Mangalore, Kasturba Medical College, Manipal, Karnataka, India; 4 Sports Performance Research Centre New Zealand, AUT University, Auckland, New Zealand

**Keywords:** Resistance training, Strength training, Exercise, 1RM, 5RM, Test-retest

## Abstract

**Background:**

Research into the kettlebell swing has increased in the last decade. There has been a paucity of literature assessing an individual’s ability to perform the kettlebell swing exercise. The purpose of this study was to determine the test-retest reliability of the one and five repetition maximum (1RM and 5RM) kettlebell swing.

**Materials & Methods:**

Twenty four recreational resistance-trained participants performed an isometric mid-thigh pull (IMTP) and two familiarization sessions followed by three test sessions for each RM load approximately one week apart, using a custom-built plate-loaded kettlebell. On each test occasion, subjects completed a series of warm-up sets followed by 3–4 progressively heavier kettlebell swings to a standardized height until 1RM or 5RM was reached. Test-retest reliability was calculated using the intra-class correlation (ICC) and typical error was represented as the coefficient of variation (CV%) with 90% confidence limits (90% CL). The smallest worthwhile change (SWC%) representing the smallest change of practical importance, was calculated as 0.2 × between-subject standard deviation. The relationship of kettlebell swing performance and maximum strength was determined by Pearson correlation with ±90% CL between the absolute peak force recorded during IMTP and 1RM or 5RM.

**Results:**

Results demonstrated a high test-retest reliability for both the 1RM (ICC = 0.97, 90% CL [0.95–0.99]; CV = 2.7%, 90% CL [2.2–3.7%]) and 5RM (ICC = 0.98, 90% CL [0.96–0.99]; CV = 2.4%, 90% CL [1.9–3.3%]), respectively. The CV% was lower than the SWC for both the 1RM (SWC = 2.8%, 90% CL [1.9–3.5]) and 5RM (SWC = 2.9%, 90% CL [1.9–3.6]) kettlebell swing. The correlation between IMTP absolute peak force and the 1RM (r = 0.69, 90% CL 0.43–0.83) was large and very large for the 5RM (r = 0.75, 90% CL [0.55–0.87]).

**Conclusions:**

These results demonstrate the stability of 1RM and 5RM kettlebell swing performance after two familiarization sessions. Practitioners can be confident that changes in kettlebell swing 1RM and 5RM performance of >3.6 kg represent a practically important difference, which is the upper limit of the 90% CL.

## Introduction

The use of kettlebells as resistance training implements has gained increasing popularity (Meigh et al., 2019). The increased popularity of kettlebells is likely due to their versatility in allowing an extensive range of exercises to be performed in order to provide a stimulus for improving a range of muscular strength, power and endurance qualities (Lake & Lauder, 2012a, 2012b; Wade et al., 2017; Wesley & Kivi, 2017). This increase in popularity coincided with the commercial range of kettlebells increasing from 4–48 kg (Tsatsouline, 2006) to 2–94 kg (Meigh et al., 2019). The most commonly researched exercise is the kettlebell swing (Meigh et al., 2019), which is commonly performed with heavier kettlebells and has been the topic of future research suggestions (Lake & Lauder, 2012b). However, despite its popularity, little data exists on assessment protocols for this movement.

In contrast to kettlebell training, valid and reliable protocols for the assessment for a range of strength and power movements such as the squat, bench press, and power clean are well established (McGuigan & Winchester, 2008; Sheppard et al., 2008). These protocols often involve the determination of the maximum load that can be lifted for a specified number of repetitions which is known as the repetition maximum (RM) (Grgic et al., 2020; Reynolds, Gordon & Robergs, 2006). In major compound lifts, RM load is commonly assessed between 1RM and 5RM but can be conducted at any RM (McMaster et al., 2014). The kettlebell swing has been prescribed with 8–12RM (Lyons et al., 2017) and a 20RM load (Sørensen et al., 2021). Additionally, less common methods have also been used such as: percentage of isometric strength (Maulit et al., 2017), loads based on body weight (Lake & Lauder, 2012a; Levine et al., 2020), expert opinion (Farrar, Mayhew & Koch, 2010), peak power (Kartages et al., 2019) and Rating of Perceived Exertion (Meigh et al., 2022). These methods can be used to guide training prescription, however, assessing RM also provides a method of assessing performance change within a chosen exercise following training interventions (McMaster et al., 2014). However, in order to do this effectively, the reliability of RM loads in specific exercises should first be established.

Reliability refers to the consistency of results between consecutive tests or performances (Comfort & McMahon, 2015; Cormack et al., 2008; Hopkins, 2000). This concept is important in strength training environments as it provides quantification of the “noise” in a test (Hopkins, 2000). Noise is the result of both biological and technical variation and can therefore be used to determine whether any change in performance can be considered practically important based on whether the inherent noise in the test has been exceeded (Appleby, Newton & Cormack, 2019).

Reliability values, in the form of the Typical Error (TE) and coefficient of variation (CV%) for common resistance training exercises at a range of RM loads have been well established (Comfort & McMahon, 2015; Cormack et al., 2008; Hopkins, 2000). For example, values for the bench press and squat are reported as <1 CV% (Seo et al., 2012), which are somewhat lower than for movements requiring higher skill levels, such as the power clean 4.8 CV% (Sheppard et al., 2008). Further, the squat correlates nearly perfectly to isometric strength (r = 0.97) (Mcguigan et al., 2010), yet the kettlebell swings relationship to isometric strength is unknown. Whilst reliability values are well known for a range of resistance training exercises, no data exists for the kettlebell swing. Therefore, the primary aim of this research is to establish the reliability of the 1RM and 5RM kettlebell swing, and the secondary aim is to determine their relationship to isometric strength.

## Materials and Methods

### Trial conditions

A within-subject test-retest design was used to determine the reliability of the kettlebell swing 1RM and 5RM in recreationally resistance-trained participants. Participants attended an isometric strength assessment, two familiarization sessions, and then three separate sessions for each of the 1RM and 5RM assessments, combining for a total of nine sessions. The order of the participant’s attendance for the 1RM and 5RM was randomly assigned. The 1RM and 5RM sessions took place 72 h apart and there was 1 week between repeat trials of the 1RM and 5RM. The participants could train normally after each RM session to avoid detraining and minimize any training interference.

### Participants

Twenty four male and three female recreationally resistance-trained participants were recruited from local gyms with at least one year of resistance and kettlebell training experience. Twenty four males (age: 31 ± 5 years; training age: 9 ± 5 years; height: 183 ± 7 cm; body mass: 91 ± 13 kg) completed the first 5RM session, however, data collection was then impeded by the COVID-19 pandemic state restrictions, limiting participation. Therefore, a total of 23 males completed the first 1RM session, and 21 and 20 males completed all three of the 1RM and 5RM sessions, respectively. Females were excluded from analysis due to low statistical power. All participants were free of any injury that would impact performance and provided written, informed consent prior to testing. Ethical approval was granted by the Australian Catholic University Human Research Ethics Committee (2018-265E).

### Procedures

All sessions included a warm-up involving 5 min of stationary cycling at a self-selected pace. Isometric strength assessment involved an isometric mid-thigh pull (IMTP). During the IMTP participants stood on portable force plates (PASCO PS-2142; PASCO 147 scientific, Roseville, CA, USA) and secured their grip with straps upon an immovable bar. When the participants were in the correct position the instruction to pull “hard and fast” was given (McGuigan & Winchester, 2008). The participants performed three trials of 5 s with 3 min rest (McGuigan & Winchester, 2008). During the five seconds vertical ground reaction force was recorded at 1,000 hz. The kettlebell familiarization sessions involved progressively heavier sets of the kettlebell swing starting with a set of 8–12 repetitions and finishing with either 4RM or 8RM for the 1RM and 5RM familiarization sessions, respectively. An estimated 1RM was calculated from the heaviest set of four repetitions during the warm-up for the 1RM familiarization trial using the following formula: ${\it Estimated}\; 1{\it RM}=\frac{\it Weight \, lifted}{1.0278-0.0278 \times {\it repetitions\, performed}}$ (Brzycki, 1993).

**Figure 1:**
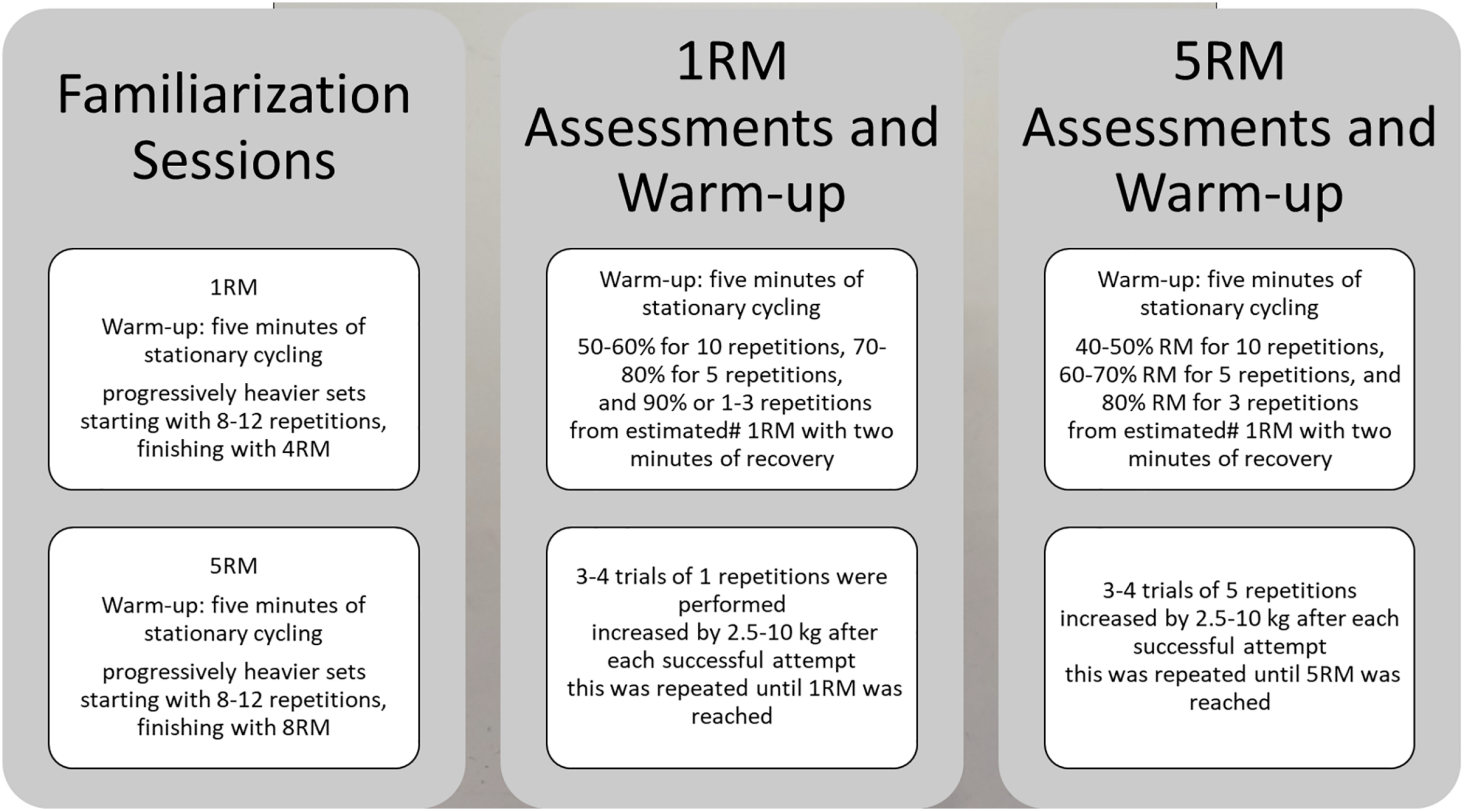
Protocols. Familiarization, 1RM and 5RM assessments and warm-up protocols.

The protocol for the reliability trial sessions is outlined in Fig. 1. Following the 5 min of stationary cycling, the warm-up sets for the 1RM sessions included 50–60% 1RM for 10 repetitions, 70–80% 1RM for five repetitions, 90% 1RM for 1–3 repetitions from estimated 1RM. The warm-up for the 5RM sessions involved 40–50% 1RM for 10 repetitions, 60–70% 1RM for five repetitions and 80% 1RM for 1–3 repetitions from estimated 1RM.

**Figure 2:**
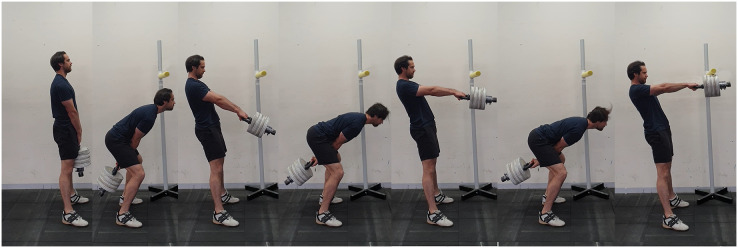
Kettlebell swing assessment. Illustrates the starting position, followed by two build up swings and a successful 1RM kettlebell swing.

Participants completed the 1RM and 5RM using the hip hinge swing style with self-selected ankle dorsiflexion to accommodate the maximum effort. The kettlebell was swung with the intention of making contact with a marker set to standing acromion process height to ensure swing height consistency, (see Fig. 2). The swing trials started in the deadlift finish position, the first and second repetitions were considered as ‘build up’ swings. The build-up swings allowed increased momentum and displacement to impose a similar eccentric phase as other repetitions. The third swing was considered the first repetition attempt for the 1RM and 5RM. A total of 5 min of rest was allowed between the RM attempts. The criterion for successful kettlebell swing attempts was that the plates of the kettlebell needed to contact the foam marker, illustrated in Fig. 2. Three to four trials of one or five repetitions were performed, with the load incrementally increased by 2.5–10 kg after each successful attempt, this was repeated until 1RM or 5RM was reached (Sheppard et al., 2008). The trial was disregarded and retested with the same load if there was a loss of balance.

### Instrumentation

An adjustable foam measuring marker was used as a target for the 1RM and 5RM assessments utilizing a plate-loaded kettlebell. The foam marker was free to move up and down the vertical beam and was secured with spring-loaded clamps. It was set to the top of the acromion process height and adjusted to accommodate the plate radius. The plate-loaded kettlebell had a 35 mm handle with a shaft that was 280 mm long and a diameter of 50 mm. The plate-loaded kettlebell was loaded with 1, 2.5, 5 and 10 kg steel plates (MA1, Dandenong South, Australia), when loaded on the shaft these plates (Fig. 2) added 12, 12, 14 and 15 mm respectively to the length of the loaded plate stack. These plates were not calibrated so the kettlebell was weighed on a portable force plate (PASCO PS-2142; PASCO scientific, Roseville, CA, USA), after the successful 1RM and 5RM trials.

### Statistical analyses

*A priori* power analysis to determine the minimum sample size (*n*) was performed with R (version 4.0.2 (2020-06-22) package ‘ICC.Sample.Size’ (Zou, 2012) (input parameters: (*p* = 0.90, *p*0 = 0.73, k = 3, alpha = 0.05, tails = 2, power = 0.80)). Twenty participants were required in order to detect excellent reliability (ICC ≥ 0.90). The test-retest reliability statistical analyses included a consecutive pairwise intraclass correlation coefficient (ICC) (3, 1) and typical error represented as the coefficient of variation (CV%) ±90% confidence limits (CL) and difference in mean using a customized Excel spreadsheet (Hopkins, 2017). The ICC was classified by the following scale: ‘poor’ ICC = <0.5, ‘moderate’ ICC = 0.5–0.75, ‘good’ ICC = 0.75–0.9, and ‘excellent’ ICC = >0.9 (Koo & Li, 2016). The smallest worthwhile change (SWC) was determined as 0.2 × between-participants SD ± 90% CL representing the smallest practically important change (Appleby, Cormack & Newton, 2019; Appleby, Newton & Cormack, 2019; Tofari et al., 2015). If the CV% was smaller than the SWC, the 1RM and 5RM tests were considered sensitive enough to detect the SWC. The relationship of kettlebell swing performance and maximum strength was determined by Pearson correlation with ±90% CL (Hopkins, 2017). The highest absolute peak force value for the IMTP and the first trial of either the 1RM or 5RM were analyzed. The Pearson correlation coefficient was classified by the following scale: ‘trivial’ r < 0.1, ‘small’ r = 0.1–0.3, ‘moderate’ r = 0.3–0.5, ‘larger’ r = 0.5–0.7, ‘very large’ r = 0.7–0.9, ‘nearly perfect’ r = 0.9–0.1, ‘perfect’ r = 1 (Hopkins, 2014).

## Results

The mean ± SD loads for each trial of the 1RM and 5RM are displayed in Table 1. Table 2 contains the CV%, ICC and SWC for the comparison of trial 1 *vs* trial 2 and trial 2 *vs* trial 3. The CV% values and ICC’s of pairwise comparisons were similar in both the 1RM and 5RM conditions. In all cases the CV% was less than the SWC. The mean ± SD of the IMTP absolute peak force was 3,555 ± 647N and there was a large (r = 0.69, 90% CL [0.43–0.83]) and very large (r = 0.75, 90% CL [0.55–0.87]) correlation between maximal isometric strength and the 1RM and 5RM swing, respectively.

**Table 1:** The 1RM and 5RM kettlebell swing mean and difference in the mean (kg).

Intensity	Trial 1 (kg) (±SD)	Trial 2 (kg) (±SD)	Trial 3 (kg) (±SD)	Mean difference trial 2–1 (kg) (±90% CL)	Mean difference trial 3–2 (kg) (±90% CL)
1RM	73.93 ± 10.52	75.53 ± 11.23	76.50 ± 10.43	1.60 [0.72–2.48]	0.97 [−0.07 to 2.01]
5RM	67.49 ± 10.01	70.67 ± 9.72	70.61 ± 9.66	3.19 [2.34–4.04]	−0.07 [−0.83 to 0.70]

**Note:**

SD, standard deviation; CL = confidence limits.

**Table 2:** Test-retest ICC, CV% and SWC% for 1RM and 5RM kettlebell swing.

Intensity	ICC (±90% CL)	CV% (±90% CL)	SWC% (±90% CL)	ICC (±90% CL)	CV% (±90% CL)	SWC% (±90% CL)
	Trial 2–1			Trial 3–2		
1RM	0.98 [0.96–0.99]	2.4 [1.9–3.3]	2.8 [1.9–3.5]	0.97 [0.94–0.99]	2.7 [2.2–3.7]	2.8 [1.9–3.5]
5RM	0.98 [0.95–0.99]	2.4 [1.9–3.3]	2.9 [1.9–3.6]	0.98 [0.96–0.99]	2.1 [1.7–2.9]	2.8 [1.9–3.5]

**Note:**

ICC, intraclass correlation coefficient; CV, coefficient of variation; SWC%, smallest worthwhile change, calculated as a 0.2 times the between-subject pure SD and represented as a percentage.

## Discussion

The primary aim of this research was to quantify the reliability of the 1RM and 5RM kettlebell swing. The results suggest that both the 1RM and 5RM kettlebell swing possess acceptable inter-day reliability following two familiarization trials. Furthermore, the CV% of the 1RM and 5RM kettlebell swing is less than the SWC, demonstrating that any variation in 1RM or 5RM performance greater than the SWC represents a practically important change.

The assessment of the maximum load that can be lifted for a specified number of repetitions is commonplace in strength and conditioning practice (Lawton, Cronin & McGuigan, 2014). Pre and post-intervention RM assessment is commonly used to determine changes in the performance of a specific exercise. However, the capacity to use this information to determine whether a practically important change has occurred from one test to the next is only possible when the variation or “noise” from both biological and technical sources has been quantified (Currell & Jeukendrup, 2008). This data is available for many common protocols across a variety of repetition ranges, including the squat, bench press and power clean (Comfort & McMahon, 2015; Grgic et al., 2020; Seo et al., 2012). In general, the “noise” appears to increase with increasing complexity of movement, but this increase is only slight (Grgic et al., 2020; Sheppard et al., 2008). Whilst the kettlebell swing may appear to be a somewhat more complex movement to assess than other multi-joint exercises, the results of the current research demonstrates similar error (ICC = 0.97, 90% CL [0.94–0.99] and CV = 2.7%, 90% CL [2.1–2.7%]) to that typically observed in both single joint exercises and more complex tasks such as the power clean (Comfort & McMahon, 2015; Grgic et al., 2020; Lawton, Cronin & McGuigan, 2014; McCurdy et al., 2008).

In addition to exercise complexity, factors such as training status, age, body–region, and sex, have been examined for their impact on reliability and in general, it appears that these factors have minimal impact (Grgic et al., 2020). The findings of this research are in agreement with previous work in terms of the impact of familiarization, as reliability remained largely unchanged when the difference in trial one *vs* trial two and trial two *vs* trial three performance is considered (Banyard, Nosaka & Haff, 2017; Grgic et al., 2020; Ritti-Dias et al., 2011; Seo et al., 2012). This suggests that a single familiarization trial is sufficient to reduce the impact of a substantial learning effect (Bridgeman et al., 2016; do Nascimento et al., 2017). Furthermore, the reliability values for both the 1RM and 5RM (ICC = 0.97–0.98, CV = 2.1–2.7%) swing are towards the top of the range reported for different exercises in a similar population (ICC = 0.64–0.99, CV = 0.5–7.8%) (Grgic et al., 2020).

An interesting finding from the current research is the similarity in reliability of the 1RM and 5RM kettlebell swing. The number of repetitions in RM tests has been shown to have little difference in the reliability between a 1RM power clean (CV = 4.8%) and a 5RM leg press (CV = 2.2–4.7%) (Gail & Künzell, 2014; Lawton, Cronin & McGuigan, 2014). Additionally, other work has shown reliability to be relatively stable across the number of repetitions in an RM test (Gail & Künzell, 2014; Lattari et al., 2020; McCurdy et al., 2004; Santos et al., 2019). The current results suggest that unless a 1RM value is specifically required, a 5RM test may be a viable option as although the test is still maximal, the absolute load will be lower and this may be important in certain populations (*e.g.*, lower training age, participants with lower kettlebell swing skill).

A useful aspect of determining test-retest reliability is that it allows calculation of the smallest worthwhile change (SWC) (Appleby, Cormack & Newton, 2019; Appleby, Newton & Cormack, 2019). The SWC represents the smallest change in performance that is likely to be of practical importance for athletic performance (Appleby, Cormack & Newton, 2019; Appleby, Newton & Cormack, 2019). There are numerous methods that have been proposed for calculating the SWC, including as a fraction (commonly 0.2) of the between participant SD (Buchheit, 2016; Datson et al., 2021). Using this method, the TE/CV% of the 1RM and 5RM kettlebell swing is less than the SWC. As a result, for a change in performance to be considered practically useful it must not only exceed the TE/CV% but in this case also exceed the SWC (Appleby, Cormack & Newton, 2019; Appleby, Newton & Cormack, 2019). The findings of this work are similar to those of previous work examining a range of exercises such as the bench press, squat and arm curls where the TE/CV% was less than the SWC (do Nascimento et al., 2017). An arguably more relevant aspect is the signal-to-noise ratio (Crowcroft et al., 2017; Ryan, Kempton & Coutts, 2020). Whilst the custom kettlebell device used in this research allowed small increases in load across repetitions (2.5 kg), and therefore a likely relatively precise determination of 1RM and 5RM values, commercially available kettlebell increments are much larger (typically 8 kg or up to 12 kg for loads >48 kg). In this case, the load increments in commercially available kettlebells can be considered the “signal” and the CV% the “noise”. The fact that the load increments often exceed the SWC means that any change in kettlebell 1RM or 5RM swing performance observed using commercially available kettlebells, represents a practically meaningful performance change.

The use of a smallest increment of 2.5 kg for RM testing in the current study was based on previous work (Sheppard et al., 2008), but has potentially resulted in a small underestimation of the true 1RM and 5RM value. However, given the increments in commercially available kettlebells typically exceed this amount, it does not impact the finding that progression in RM performance from one kettlebell load to the next represents a practically important change.

Additionally, with a plate-loaded kettlebell as used in this study, the center of mass (COM) changes with each load increment resulting in an overload from both the increased mass and increased distance of the kettlebell COM from the fulcrum (Serway & Jewett, 1998). As both these factors will independently increase the required muscular force to complete the kettlebell swing, they should be quantified. The kettlebell COM can be calculated with the following equation where; m = mass and x = meters: COM = (m_1_x_1_ + m_2_x_2_ + m_3_x_3_)/(m_1_ + m_2_ + m_3_) (Serway & Jewett, 1998). Further, commercially available plate loaded kettlebells can be loaded with either a distal to proximal configuration, or a proximal to distal configuration from the handle. Care should be taken to avoid the use of interchangeable plate loading order. It is possible that RM loads were a function of changes to both the mass and its distribution and therefore that the results may have been altered with a kettlebell with different mass distribution.

In contrast to a kettlebell swing with a plate-loaded kettlebell, 1RM barbell exercise assessments may be more suitable for testing maximum strength. Despite the 1RM being considered the gold standard method of field-based testing for maximal strength, the kettlebell swings relationship to maximal isometric strength is lower than other exercises currently used for this purpose. For example, the barbell squat (r = 0.86–0.97) (Bazyler, Beckham & Sato, 2015; Mcguigan et al., 2010), deadlift (r = 0.88) (De Witt et al., 2018), snatch (r = 0.83), and the clean and jerk (r = 0.84) (Beckham et al., 2013), all have a stronger relationship with isometric strength compared to the values reported for the 1RM and 5RM kettlebell swing in the present study. Therefore, these other exercises offer better validity for field-based tests to assess maximum strength.

## Conclusions

This research demonstrates that both the 1RM and 5RM kettlebell swings possess excellent reliability. Critically, the SWC is less than the TE/CV% in both tests. Furthermore, both these values are lower than commonly available kettlebell increments. Further research using smaller increments during RM testing may allow more precise estimation of reliability and the SWC. Practitioners can be confident that assessment of 1RM and 5RM kettlebell performance following two familiarization sessions is not prone to large error. Due to the fact that commercially available kettlebell increments generally exceed the CV% and SWC demonstrated in this research (*i.e.*, signal > noise) (Appleby, Newton & Cormack, 2019), changes in performance based on an increase or decrease in 1RM or 5RM kettlebell swing performance can be considered practically meaningful. Future research could determine the effect of plate configuration upon biomechanical, physiological and perceptual characteristics of the swing as well as the resulting adaptations such training may reduce. Additionally, accurate knowledge of RM values may allow more precise training prescription (*e.g.*, % based loads), which could better elucidate the kettlebell swing’s optimal training zones for strength and power adaptation.

## Practical application

Barbell exercises have a stronger relationship with isometric strength and are therefore a better assessment of maximum strength. In contrast, the 1RM or 5RM swing is best used to assess swing performance pre and post-training intervention. Practitioners may also wish to consider plate loaded rather than fixed load kettlebells to allow more precise RM determination. Further, if using a plate loaded kettlebell, plates of the same mass should have the same width to ensure that the COM progressions are standardized. Plate loaded kettlebells should be loaded in the same way, either distal to proximal from the handle or proximal to distal from the handle. Finally, a 5RM kettlebell swing may represent a useful alternative to a 1RM for lesser trained individuals.

## Supplemental Information

10.7717/peerj.14370/supp-1 Supplemental Information 1 Data and code.

10.7717/peerj.14370/supp-2 Supplemental Information 2 Swing Protocol.

## Additional Information and Declarations

### Data Availability

The following information was supplied regarding data availability: The raw data is available as a Supplemental File.
