# Supplementary material for: Reliability of kettlebell swing one and five repetition maximum
Source: PeerJ. 2022 Nov 21;10:e14370. doi: 10.7717/peerj.14370 (PMC9686413; doi:10.7717/peerj.14370)
Supplement: Supplemental Information 2 [file peerj-10-14370-s002.docx]

**Trial protocol.**

**Trial timeline:**

**Figure 1.** Session within the same week had approximately 72 hours between them.

**Testing session protocol warm-up**

Following the five minutes of stationary cycling, the warm-up sets of for the 1RM sessions included 50-60% 1RM for 10 repetitions, 70-80% 1RM for 5 repetitions, 90% 1RM for 1-3 repetitions from estimated 1RM. The warm-up for the 5RM sessions involved 40-50% 1RM for 10 repetitions, 60-70% 1RM for 5 repetitions and 80% 1RM for 1-3 repetitions from estimated 1RM.

**Assessments protocol**

The swing trials started in the deadlift finish position, the first and second repetitions were considered as ‘build up’ swings. The build-up swings allowed increased momentum and displacement to impose a similar eccentric phase as other repetitions. The third swing was considered the first repetition attempt for the 1RM and 5RM. Five minutes of rest was allowed between the RM attempts. The criterion for successful kettlebell swing attempts was that the plates of the kettlebell needed to contact the foam marker, illustrated in figure 2. Three to four trials of one or five repetitions were performed, with the load incrementally increased by 2.5-10 kg after each successful attempt, this was repeated until 1RM or 5RM was reached.


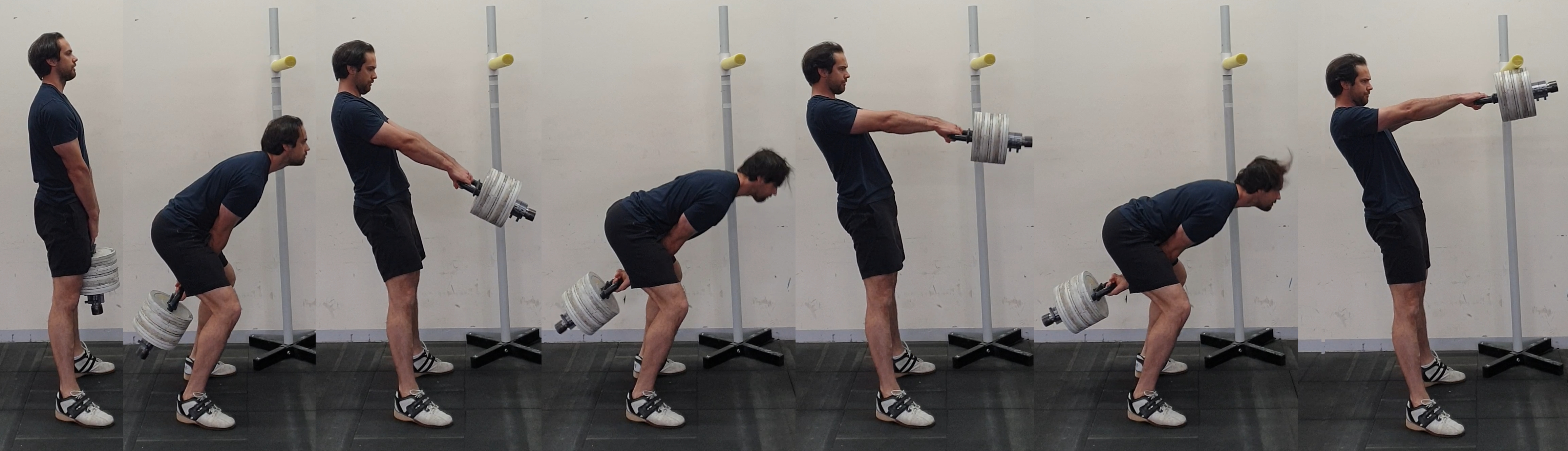


**Figure 2.** Illustrates the starting position, followed by two build up swings and a successful 1RM kettlebell swing
